# Reintroducing community health psychology: A journal‐based analysis of the visibility and integration of community‐based participatory research

**DOI:** 10.1111/aphw.70185

**Published:** 2026-07-09

**Authors:** Drexler James, Harrison J. Schmitt

**Affiliations:** ^1^ Department of Psychology University of Minnesota Twin Cities, Minneapolis Minnesota USA; ^2^ Department of Psychology Skidmore College Saratoga Springs New York USA

**Keywords:** community‐based participatory research, community health psychology, participatory action research

## Abstract

Community‐based participatory research (CBPR) provides a collaborative framework for addressing health inequities through equitable partnerships, community engagement, and culturally responsive methods. Although widely used in public health and community psychology, CBPR's visibility within the core publication outlets of health psychology remains unclear. This review, inspired by the 2004 *Journal of Health Psychology* special issue on Community Health Psychology, examines the extent to which CBPR is represented and integrated within the institutional core of health psychology journals over the past two decades. We systematically searched for CBPR‐related terms in 10 health psychology journals (e.g., *Applied Psychology: Health and Well‐being*, *Journal of Health Psychology*) between 2004 and 2024. From the empirical articles identified, we determined whether CBPR was mentioned, discussed, or substantively implemented. For studies using CBPR methods, we coded engagement with nine core elements (e.g., community involvement in design). Of the 14,084 articles published between 2004 and 2024, 28 empirical articles mentioned CBPR without elaboration (~0.2%), 16 discussed CBPR concepts without implementing participatory methods (~0.1%), and 30 incorporated CBPR methods (~0.2%). Among studies incorporating CBPR, engagement varied considerably: on average, studies included 2.67 CBPR elements (*SD* = 2.99), with scores ranging from 0 to 8; the largest subset (*n* = 10; 33.3%) addressed only one element. These findings indicate a marked disconnect between the field's stated commitments to equity and the visibility of participatory approaches within its primary publication infrastructure. We conclude by identifying structural and conceptual factors that may limit CBPR's integration and outlining directions for advancing participatory approaches within health psychology.

## INTRODUCTION

Community‐based participatory research (CBPR) has offered a prominent framework for conducting community‐centered research that addresses health disparities (see Suarez‐Balcazar et al., 2020). Core elements of CBPR—sometimes referred to as participatory action research—include involving a given community in all phases of the research process, maintaining equitable relations among researchers, participants, and partners, ensuring cultural sensitivity in research methods and pursuing long‐term sustainability of context‐specific interventions (Collins et al., 2018; Wallerstein & Duran, 2010). A variety of recent systematic reviews and meta‐analyses have demonstrated that CBPR may be effective at increasing participation in community health interventions (Riccardi et al., 2023), improving mental and physical health outcomes (Halvorsrud et al., 2021; McFarlane et al., 2024), and encouraging change in health behaviors (McCuistian et al., 2023; Queral et al., 2024), particularly in diverse and disadvantaged communities that are often excluded from health research.

In writing the present paper, we were inspired by a particular special issue published in 2004 in the *Journal of Health Psychology* entitled “Community Health Psychology” (Campbell & Murray, 2004). This special issue, which includes 10 articles now collectively cited 1050 times, sketches the values and assumptions of a community health psychology, provides examples of participatory research to address health disparities, and envisions a path toward a culturally sensitive and justice‐oriented sub‐discipline within health psychology. CBPR has been widely adopted in adjacent fields such as community psychology and public health (Suarez‐Balcazar et al., 2020; Wallerstein & Duran, 2010). However, the extent of its integration into mainstream health psychology remains largely unexamined, even in light of the special issue published 20 years ago.

Given this, we sought to explore whether, and to what extent, participatory methods and community‐engaged approaches have been embraced within mainstream health psychology over the past two decades. Two main questions guided the present paper: (a) To what extent has the field of health psychology adopted participatory approaches? and (b) If the field has not adopted such approaches, how might we envision a more participatory and community‐centered health psychology moving forward? To explore these questions, we introduce the basics of CBPR to health psychology readers then use systematic review methods to quantify the extent to which CBPR has been used in the field. We then present strategies (re)introducing CBPR into mainstream health psychology research and discuss some ways in which health psychologists adopt CBPR values and approaches in their scholarship.

### What is community health psychology?

The field of community psychology has long been guided by dual motivations to understand individuals as nested within community contexts and to intervene at the community level on those aspects of these contexts that inhibit human flourishing (Trickett, 2009). Campbell and Murray (2004) argue for a branch of this field called community health psychology as an area of study “concerned with the theory and method of *working with communities* to combat disease and to promote health” (p. 187, emphasis added). They also explicitly see community health psychology as being a response and challenge to the perceived inadequacies of mainstream health psychology research at the time. These inadequacies included the “minimal contributions” that the field had made to understanding the root causes of health inequities, acknowledging the broader sociocultural contexts in which health behavior and illness occur, and producing health psychology interventions with real‐world efficacy. For us, we see one of the key tenets of the community health psychology they envisioned in this special issue as a *critical engagement with CBPR in health psychology research*. Indeed, CBPR is central to the mission of a community health psychology because it serves multiple ends that work in combination to address health inequities. Still, we feel that a robust tradition of CBPR in health psychology has yet to be realized—which motivated our current inquiry.

### Why community‐based participatory research?

There is a rich and diverse history to the CBPR tradition. A “Northern” tradition rooted in Kurt Lewin's (1946) “action research” in psychology and a more politically revolutionary “Southern” tradition rooted in Paulo Freire's *Pedagogy of the Oppressed* (2005) and the many Latin American research traditions that stemmed from this work (e.g., Liberation Psychology, Martín‐Baró, 1994; the sociological “science of the people,” Fals‐Borda, 1981) have both influenced contemporary CBPR research (Macaulay, 2017). From a fringe orientation in the social and health sciences, it has now become a far more prominent and heterogenous orientation to research and community collaboration over the past 30 years. Some have critiqued this trajectory for the ways it has strayed from its more radical roots and been coopted by state and private institutions as a research approach that complements traditional mainstream approaches (Jordan & Kapoor, 2016; Stoecker, 2003). According to Israel et al.'s (1998, 2026) numerous reviews of this area, CBPR is used in a variety of different ways that range across a continuum from research that is merely conducted *in* community contexts to research that intimately *involves* the community in the research process, to research that is *entirely community driven*. As an umbrella term, CBPR thus incorporates approaches that exist along two orthogonal continua: from approaches that seek radical transformation of society to those that seek amelioration of community concerns through research (without radical transformation) on one axis, and research that is more or less driven by the community on the other axis.

Keeping this varied history and the various approaches currently adopted in mind, we build on Wallerstein et al.'s (2017) general definition of CBPR as “a collaborative approach to research that equitably involves all partners in the research process and recognizes the unique strengths that each brings. CBPR begins with a research topic of importance to the community with the aim of combining knowledge and action for social change to improve community health and eliminate health disparities” (p. 4). CBPR should thus be considered not a “method” or a set of methods per se, but an orientation to conducting research that centers power sharing with communities that affords the use of a constellation of different methods. Proponents of CBPR argue for its effectiveness, sensitivity to cultural contexts, ability to adapt interventions to real‐world settings, and its potential to bring about broader social change alongside individual‐level behavior change. Before assessing the extent to which CBPR has been taken up in health psychology, we first outline the core values and assumptions of a more community‐centered approach to the field.

#### Core assumptions and values

Although CBPR is by no means a monolith (Israel et al., 1998; Stoecker, 2003), we present four core assumptions and values that tend to guide much of the research in this area: (a) alternative epistemologies such as critical or constructivist epistemologies, (b) an ecological model of health and health behavior, (c) methodological pluralism, and (d) a commitment to social–structural change (alongside individual–behavioral change). We introduce readers to each of these core values hereafter—they are also summarized in Table 1.

**TABLE 1:** Core values of community‐based participatory research (CBPR) for health psychology.

Value	Definition	Implications for community health psychology research	Further reading
Alternative epistemologies	In contrast to the logical empiricist epistemology, community heath psychologists may adopt alternative epistemologies that value the ways of knowing of “non‐experts” (e.g., social constructivist epistemologies), that critique mainstream scientism, and that seek to change the social–structural status quo (e.g., critical and decolonial epistemologies)	Value multiple ways of knowing, and so use multiple methods to generate knowledge Research is (often) explicitly critical of both mainstream approaches and of social structures that produce inequities Favors a politicized stance over a value‐neutral stance	Murray (2012): “Critical health psychology and the scholar–activist tradition” Reyes Cruz (2008): “What if i just cite Graciela? Working toward decolonizing knowledge through a critical ethnography” Rasmus (2014), “Indigenizing CBPR”^a^
Ecological model of health	In contrast to biomedical, clinical, or biopsychosocial models of health, community heath psychologists may use an ecological model to understand health and to identify loci for intervention. Under such a model, individual health, illness, and wellness are nested within communities and within broader historical, cultural, political, and geographical contexts, all of which function in mutually constitutive ways that shift dynamically with time	Interventions focus on community‐ and structural‐level changes (e.g., policy, community empowerment) to address health Focus is on social and structural determinants of health (behavior) that exist upstream from individuals	Lounsbury and Mitchell (2009): “Special issue on social ecological approaches to community health research and action” Allen et al. (2019): “A protective factors model for alcohol abuse and suicide prevention among Alaska Native youth”^a^ Allen et al. (2019): “Strengths‐based assessment for suicide prevention”^a^
Methodological pluralism	In seeking culturally situated and critical accounts of health, community health psychologists use multiple and mixed methods. This includes a variety of quantitative and qualitative approaches, often employed in a participatory manner	Methods are selected that best fit a given situation, community, or problem, and these methods are negotiated, selected, and enacted in collaboration with a community	Smith (1999): *Decolonizing methodologies: Research and indigenous peoples* Stephens (2008): “Conducting community health psychology,” in *Health promotion: A psychosocial approach* Rasmus et al. (2014): “Creating Qungasvik (a Yup'ik intervention ‘toolbox’)” ^1^
Commitment to social‐structural change and community empowerment	Although individual health is important for community health psychologists, the prevailing assumption is that individuals cannot flourish in unjust contexts.	A primary goal of community health psychology is to change structures, policies, and contexts, often in part through community empowerment achieved through participatory research	Murray and Poland (2006): “Health psychology and social action” Stoecker (2003): “Community‐based research: from practice to theory and back again” Rasmus et al. (2019): “With a spirit that understands: reflections on a long‐term community science initiative to end suicide in Alaska”^a^

^a^ These articles are part of a longstanding CBPR project that has sought to prevent suicide and promote wellness in Yup'ik villages in Alaska, serving here as an excellent case study in applying the values of CBPR.

##### Alternative epistemologies

As with the field of psychology at large, the mainstream epistemology in the field of health psychology involves using logical empiricism to generate knowledge about the world (Rogers, 1996). This has resulted in the field seeing experimentation in general—and the randomized‐controlled trial in particular—as the “gold standard” for the methods of understanding and intervening on health‐related variables in a somewhat objective and bias‐free manner. Although CBPR approaches do not necessarily eschew the merits of such methods, they tend to approach research from alternative epistemological (and by extension methodological) grounds. Many prominent practitioners of CBPR suggest that common alternative epistemologies include a social constructivist or a critical epistemology (Israel et al., 1998; Murray et al., 2004). The social constructivist paradigm is based around the idea that knowledge and the processes that produce it are always historically, socially, and culturally constructed. The critical epistemological approach assumes that our shared reality is shaped by powerful interests and inequalities that stem from racism, sexism, classism, ableism, and so on, and that research should challenge the social–structural status quo. These alternative epistemological grounds for CBPR provide the foundation for the other core values and assumptions of this paradigm.

##### Ecological model of health and health behavior

CBPR typically favors an ecological model of health rather than the traditional biomedical, clinical, or biopsychosocial models of health that have dominated health psychology. Although more recent trends in health psychology have seen greater attention paid to social structures and culture, a significant portion of mainstream research in the field still firmly approaches health and illness at the individual level of analysis (Hatala, 2012; Morison & Gibson, 2025; Stam, 2000). CBPR encourages a more genuinely ecological approach to health by prioritizing the community as the core level of analysis while also critically engaging with the dynamic interactions among macro‐, meso‐, and micro‐level factors (e.g., the individual embedded in a community which is embedded in a broader cultural–historical–geographical context; Collins et al., 2018; Murray et al., 2004). The target, then, of interventions produced by such a paradigm is not solely the individual's behaviors and attitudes. Because such individual‐level factors are seen as mutually constitutive of broader social forces, CBPR often targets both individual‐ and community‐ or policy‐level intervention (e.g., Estabrooks et al., 2008). This means that the focus of a community health psychology that uses CBPR is on *preventing* ill‐health through structural change and *promoting* health through sustainable community empowerment and capacity building: “Wellness cannot thrive in conditions of inequality and injustice” (Prilleltensky & Prilleltensky, 2003, p. 276; see also Stephens, 2008).

##### Methodological Pluralism

The alternative epistemological groundings of CBPR also give way to methodological pluralism. Where the empiricist approach of hegemonic health psychology favors quantitative and experimental methods, the critical and constructivist approaches in CBPR favor qualitative and participatory methods (Stephens, 2008). CBPR researchers believe that researchers trained in traditional graduate programs with a focus on the scientific method and quantitative statistical procedures are experts *only* in their field's hegemonic ways of knowing. As such, CBPR employs methods that center the subjective, culturally situated lived experience of a given illness or health disparity. These often include standard qualitative methods (e.g., interviews, focus groups, oral history; Moreno Ramírez, 2020; Rasmus et al., 2019) and more radical non‐traditional methods (e.g., photovoice, counter‐mapping, counter‐storytelling; Hergenrather et al., 2009; Hunt & Stevenson, 2017; Petteway, 2022). At times, these qualitative methods are employed in combination with quantitative methods that are more familiar to health psychologists (e.g., experiments, correlational studies; Schmitt et al., 2022). Most importantly for CBPR, these methods are employed in a participatory manner: the community members themselves are critically involved in decisions around the research questions asked, the methods used, and the analysis and interpretation of the results (especially for qualitative data; Cashman et al., 2008). Thus, CBPR often uses a mix of research methods and employs them based on horizontal, equitable input from communities that are facing a given health concern, which contrasts sharply with the often top‐down, researcher‐driven approach in mainstream health psychology.

##### Commitment to social–structural change

Finally, a core value in CBPR is to seek not just the advancement of health (behavior) for a group of individuals but to push toward broader social change (e.g., policy change, building capacity for community organizing). Although many health psychologists are well‐intentioned researchers seeking to promote health and well‐being, some more critically minded community health psychologists argue that the business‐as‐usual approach in health psychology does little to address the root causes of health disparities and merely attunes individuals to the unjust status quo through psychological and behavioral change (Jimenez & Schmitt, 2024; Rogers, 1996). By encouraging grassroots organization, participation in the production of knowledge, and social action, CBPR attempts to advance individual health and well‐being in part through social–structural changes (Murray & Poland, 2006; Stoecker, 2012; Wallerstein & Duran, 2010). For example, where a more traditional health psychology approach to environmental injustice from lead exposures might employ an intervention targeting behavior change to reduce exposures, a CBPR approach geared toward social structural change might involve community partners in policy education and advocacy in order to pressure local officials to implement routine testing and other prevention strategies (e.g., Minkler et al., 2008). Thus, CBPR researchers often eschew value‐neutrality in favor of an overt commitment to achieving social justice through participatory research and praxis.

In seeking social–structural change, one of the primary goals of CBPR is to work toward empowering a community to understand their own health, the factors inhibiting health, and the paths toward achieving health equity through collective action. For Campbell and Murray (2004, see also Smith, 1999), this process is often based on the work of Paulo Freire's notion of *conscientização* (or conscientization): the process of co‐learning to “perceive social, political, and economic contradictions, and to take action against the oppressive elements of reality” (Freire, 2005, p. 35). In a Freirian approach to CBPR, conscientization is achieved through the “dialogue” that researchers engage in with community members throughout the research process. This dialogue allows researchers and community members to simultaneously uncover the hidden, upstream oppressive forces that produce ill‐health and to co‐imagine the paths toward health equity (Smith, 1999; Taliep et al., 2022), instilling hope, shoring up networks of solidarity, and planting the seeds of long‐term grassroots activist movements.

#### What does CBPR add to traditional health psychology research?

Working directly with community members to conduct descriptive research and to design and implement interventions has much to add to the business‐as‐usual paradigms used in health psychology. Health psychologists commonly follow models such as the NIH Stage Model for Behavioral Intervention Development (Onken et al., 2014). We note that other translation and implementation models are certainly used by health psychologists (e.g., PRECEDE–PROCEED model, ORBIT model, Czajkowski & Hunter, 2021; Czajkowski et al., 2015), which vary in the extent to which they center community engagement. The NIH stage model is merely a common and representative approach adopted in the field. This cyclical model begins with basic research to inform an intervention (Stage 0), initial creation of an intervention (Stage I), efficacy testing of the intervention in research settings (Stage II), efficacy testing in community settings focused on internal validity (Stage III), effectiveness testing in community settings focused on external validity (Stage IV), and finally, implementation and dissemination of the intervention (Stage V).

The reality of much research in health psychology is that it favors Stages 0–III (Czajkowski & Hunter, 2021). Relatively few studies attempt to move basic research into community settings with non‐experimental research or methods that lack the kind of laboratory control valued in the mainstream health psychology approach (e.g., Beedie et al., 2016; Hagger & Weed, 2019). This means that much intervention research in community settings (Stage III) is performed with highly controlled research methods, which may demonstrate efficacy of an intervention only in “real‐world settings” that do not resemble the messy, uncontrolled contexts of people's actual lives. CBPR offers, at minimum, a chance to more thoroughly engage with the nuances and cultural constructions that characterize people's actual lived experiences. With these nuances at the fore from the very beginning of the “basic” research process, interventions can be designed in ways that are sensitive to the embeddedness of individuals within their historical, political, cultural, and geographical contexts (Bogart & Uyeda, 2009).

More importantly, however, is the way that CBPR injects more equitable researcher–community relations into the process of research and intervention development. Whereas the NIH stage model does not preclude community involvement at any stage, the process is still almost entirely top‐down and researcher driven. Many calls to move health psychology beyond Stages 0–III acknowledge community involvement as a means of enhancing the efficacy of interventions (Byrne, 2019; Czajkowski & Hunter, 2021; Hagiwara et al., 2020), suggesting that many health psychologists are interested in community‐based research. However, these calls rarely discuss the merits of participatory methods explicitly and often treat community involvement as an afterthought, as only relevant at the later stages, or as a supplement to the mainstream top‐down approach.

The hidden assumption of such models is that science‐trained researchers know best what questions to ask, what health and well‐being ought to look like, and how to encourage behavioral change to achieve health and well‐being. Such approaches to research reproduce unjust power imbalances between the relatively privileged researchers and the often‐marginalized communities who face health disparities (Cameron et al., 2025). Moreover, the hierarchical model of research in mainstream health psychology may unintentionally devalue the ways of knowing in a given community, potentially adding to the multiple injustices that such communities face—particularly “epistemic injustice” (Bhakuni & Abimbola, 2021; Okoroji et al., 2023).

Beyond increasing the cultural sensitivity of interventions, CBPR may advance health equity in broader and more sustainable ways. According to Stoecker (2012), the participatory nature of CBPR results in a research praxis that can “build up people's capacity to both create knowledge, and to put knowledge into practice in ways that enhance their power to win on issues and influence the course of world events” (p. 92). Power is thus wrested from the privileged researchers who acquire funding to test their own theories *on* communities, and is placed squarely back with the communities themselves, potentially contributing to sustainable community‐level change moving forward.

### CBPR in (health) psychology research

If CBPR holds the potential to advance equity, cultural relevance, and epistemic justice in health psychology, it is essential to assess the extent to which it has been taken up within the field. One effort to do so comes from a systematic review by Rodriguez Espinosa and Verney (2021), who examined CBPR publications from 2004 to 2014 across psychology and non‐psychology journals. Of the 1912 CBPR articles identified, only 16% (*n* = 311) were classified as “psychology related”—defined as either being published in a psychology journal or having at least one psychologist as an author. However, the vast majority of these psychology‐related CBPR articles appeared outside of psychology journals, with only 21% published in mainstream psychological outlets. Notably, 42% of those were concentrated in a single journal, the *American Journal of Community Psychology*. A small subset (12–15%) appeared in health behavior, health promotion, or public health journals, thereby highlighting some relevance for health psychology, although the review did not focus on this subfield explicitly.

One conclusion that can be drawn from this recent review is that psychology has made substantial contributions to CBPR studies, but this has primarily been performed by psychologists publishing in non‐psychology journals. This pattern suggests that while psychologists are contributing to CBPR scholarship, mainstream psychology journals have not served as the primary outlets for this work, limiting its visibility within the discipline. However, the extent to which this is also true for health psychology specifically remains unclear. To address this gap, we extend the work of Rodriguez Espinosa and Verney (2021) in several important ways.

First, their review, while valuable, was intentionally broad, surveying the entire psychology discipline and drawing from both psychology and non‐psychology journals. In contrast, we focus specifically on health psychology journals. Second, our review also addresses several methodological limitations of the prior study. For instance, we employed a broader search strategy that went beyond the two terms used previously (“community‐based participatory research” and “CBPR”), incorporating alternative descriptors such as “action research,” which may capture work that draws on the “Southern” traditions in the field described earlier. We also examined a longer time span (2004–2024), allowing for an analysis of longitudinal trends over two decades. In addition, whereas the previous review identified studies using CBPR methods, it did not assess the degree to which core CBPR principles were incorporated. Our analysis explicitly codes for engagement with nine CBPR elements (e.g., equitable partnerships, shared decision‐making, dissemination of findings to communities).

### Current study

Despite health psychology's emphasis on addressing health disparities and promoting equity, little is known about the extent to which the field has adopted research approaches that center community engagement and co‐creation of knowledge. Community‐based participatory research offers a collaborative, equity‐oriented framework well‐aligned with these goals. We viewed the *2004 Journal of Health Psychology* special issue on community health psychology as a possible inflection point for CBPR uptake within the field. Importantly, the present study does not aim to characterize the full scope of CBPR across health‐related disciplines or all research relevant to health psychology. Rather, it focuses on how CBPR is represented within the institutional and disciplinary core of health psychology, as reflected in its primary publication outlets.

To examine this question systematically, we conducted a bounded, discipline‐focused review of 10 journals representing the institutional core of health psychology published between 2004 and 2024. This design enables an analysis of CBPR's visibility and integration within mainstream publication channels of the field, rather than an estimate of its overall prevalence across the broader, interdisciplinary landscape in which CBPR is more widely established. Our aim was to assess whether CBPR has been taken up and how deeply its principles have been integrated into research published within these outlets.

To this end, we identified empirical articles that referenced, discussed, or implemented CBPR or related participatory approaches. For studies implementing CBPR, we coded for nine core CBPR elements—including community involvement in study design, data collection, analysis, and dissemination—to evaluate the depth of engagement. By distinguishing between mention, conceptual engagement, and methodological implementation, this approach allows us to differentiate between the visibility of CBPR as an idea and its uptake as a research practice within the field's core publication infrastructure.

## METHOD

### Transparency and openness

The search strategy, data extraction plan, and analytic plan were pre‐registered. We adhered to the Preferred Reporting Items for Systematic reviews and Meta‐Analyses (PRISMA) guidelines (Tricco et al., 2018). The completed PRISMA checklist is available in Table S1. The pre‐registration and all associated materials are available in the Online Supporting Information at https://tinyurl.com/yc6v92ee. As the unit of analysis was papers, not individuals, and no personal data were collected, no informed consent or ethical approval was necessary.

### Search strategy

We conducted a systematic search of empirical papers published between January 1, 2004 and June 30, 2024, within a predefined set of peer‐reviewed journals identified through the journal selection protocol described hereafter. The time frame was selected to begin in 2004, corresponding to the publication of a special issue on community health psychology in the *Journal of Health Psychology*, and extended through June 2024 to capture two decades of empirical research and recent developments in the field.

### Journal selection

Journal selection followed a predefined, externally anchored, and reproducible protocol designed to identify journals representing the institutional core of health psychology. Specifically, the sampling frame was restricted to journals formally affiliated with, published by, or closely aligned with major professional organizations in health psychology, including the *International Association of Applied Psychology*, *American Psychological Association*, the *European Health Psychology Society*, and the *British Psychological Society* (Division of Health Psychology). Journals were eligible for inclusion if they met all of the following criteria:

1. Institutional Affiliation: The journal is formally affiliated with, published by, or closely associated with one of the above organizations or their health psychology divisions.
2. Disciplinary Scope: The journal's stated aims and scope focus on *psychological* processes in health, illness, or health care.
3. Empirical Orientation: The journal regularly publishes peer‐reviewed empirical research using quantitative, qualitative, or mixed‐methods approaches.
4. Temporal Coverage: The journal was continuously active and indexed across the study period (2004–2024).

Applying these criteria yielded the following journals: *Health Psychology*; *Stigma and Health*; *Journal of Health Psychology*; *Psychology and Health*; *Psychology, Health, and Medicine*; *Applied Psychology: Health and Well‐being*; *European Journal of Health Psychology*; *British Journal of Health Psychology*; *International Journal of Clinical and Health Psychology*; and *Journal of Occupational Health Psychology*.

This sampling strategy reflects a deliberate analytic distinction between the production of CBPR knowledge and its disciplinary visibility. Although CBPR is widely conducted in adjacent fields such as public health, behavioral medicine, and community psychology, the present study does not aim to capture the full corpus of CBPR research relevant to health. Instead, it examines the extent to which CBPR is represented within journals that define the institutional and epistemic center of health psychology.

Journals that did not meet these criteria were excluded. This included interdisciplinary or adjacent outlets (e.g., *Social Science & Medicine*, *Annals of Behavioral Medicine*), which, although highly relevant to health psychology, are not formally affiliated with the professional organizations used to define the sampling frame and therefore fall outside the institutional scope of the present analysis. In addition, many such outlets prioritize broader social, epidemiological, or biomedical determinants of health rather than the explicit investigation of psychological processes as primary explanatory targets.

### Search terms

To identify relevant articles in each journal during the specified time frame, we conducted targeted searches using journal‐specific indexing within PsycINFO and, when necessary, through individual journal websites or databases with journal‐specific filters (e.g., PubMed, Web of Science). We used the following search terms: (a) “community‐based participatory research,” (b) “CBPR,” (c) “action research,” (d) “participatory action research,” (e) “participatory research,” (f) “community‐engaged research,” and (g) “community‐engaged action research.” These terms were chosen to reflect the various and evolving vocabulary used to describe CBPR and related participatory approaches. Detailed search procedures, including Boolean operators and journal‐specific filters, are provided in the Supporting Information.

### Inclusion and exclusion criteria

We included empirical studies, defined as investigations that involve the systematic analysis of data to generate or evaluate evidence, regardless of whether those data were newly collected or drawn from existing sources. Eligible studies employed quantitative, qualitative, or mixed‐methods approaches and provided analyzable data (e.g., numerical, textual, or observational) linked to a research question or evaluative aim. To be eligible, studies also had to substantively engage with CBPR methodology. Engagement with CBPR was operationalized using a graded classification scheme distinguishing levels of mention, discussion, and use (see succeeding text). We excluded non‐empirical publications (e.g., literature reviews, theoretical papers, commentaries, editorials) that do not involve the analysis of data, in order to focus on the practical integration of CBPR within empirical health psychology research.

### Study screening and eligibility assessment

**FIGURE 1:**
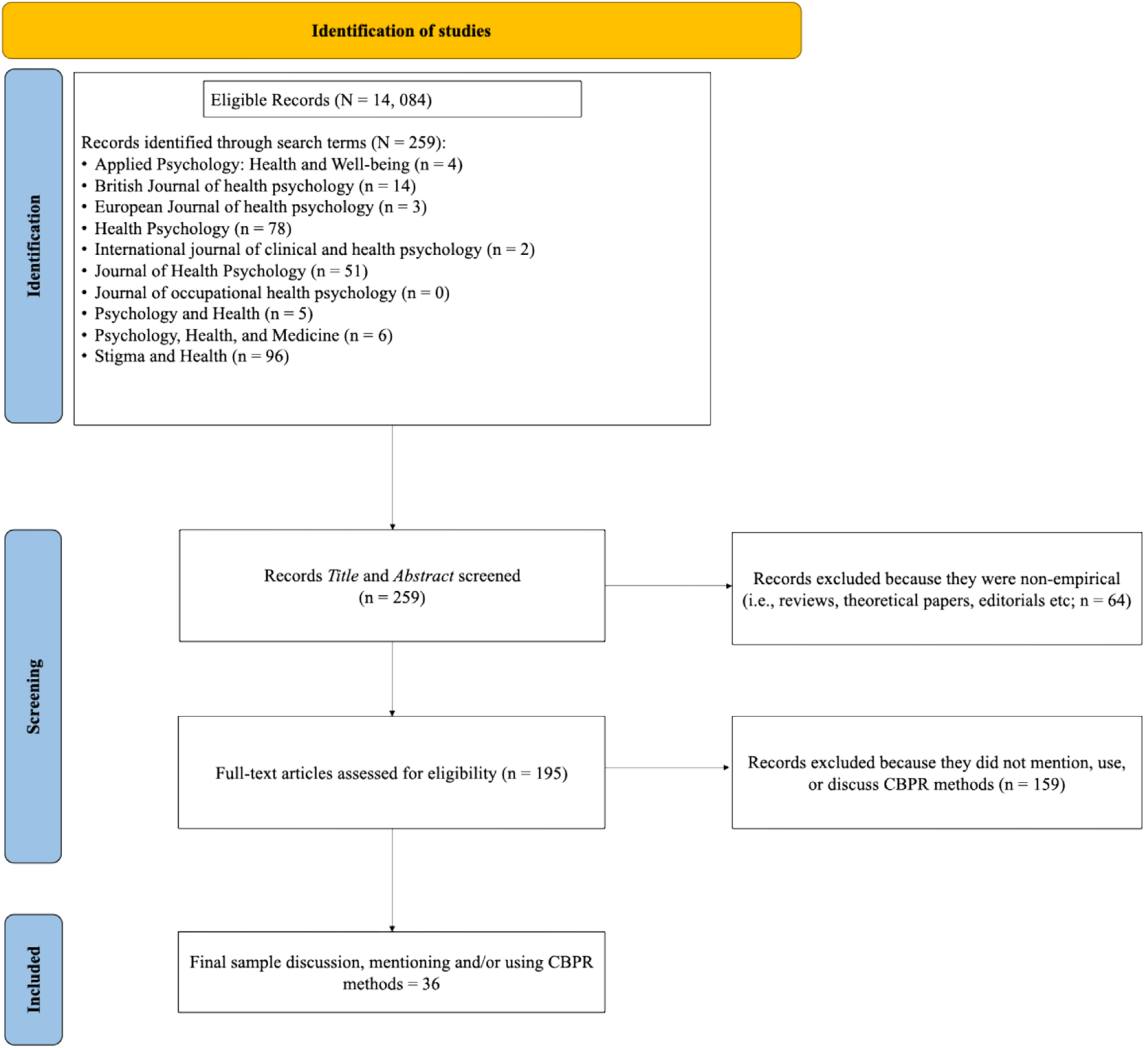
PRISMA flow diagram depicting the systematic selection process of reports included in the review. PRISMA, Preferred Reporting Items for Systematic reviews and Meta‐Analyses.

A PRISMA flow diagram summarizing the screening and inclusion process is presented in Figure 1. We uploaded the database search results in Research Information Systems (RIS) format to Rayyan.ai (Ouzzani et al., 2016), a web‐based platform designed to facilitate systematic review screening. In the initial screening phase, we reviewed titles and abstracts to identify empirical articles for potential inclusion, excluding non‐empirical publications.

To ensure consistency in the application of our inclusion criteria, both authors independently screened a random 10% subset of the retrieved records. Inter‐rater agreement for this subset was perfect (Cohen's *κ* = 1.00), and no discrepancies were identified. The remaining records were then divided equally, with each author independently screening approximately half of the remaining titles and abstracts. All studies that passed this initial screening phase were subsequently subjected to full‐text review. Because our coding process required in‐depth examination of methods sections, we conducted full‐text screening and data extraction concurrently.

We employed two complementary approaches to full‐text screening. First, we used the “Find” function in each PDF to search for predefined CBPR‐related terms corresponding to the database search terms. If one or more of these terms appeared anywhere in the article, excluding the reference section, the study was retained for further evaluation. Second, we conducted a manual review focused primarily on the methods and—where applicable—the “current study” or “approach” sections to assess whether the study reflected core features of participatory research.

The screening process unfolded in two phases. In the first (training) phase, both authors independently screened and extracted data from a random sample of 10 full‐text articles. Three coding discrepancies were identified and resolved through discussion. Inter‐rater reliability for inclusion decisions during this phase was high (Cohen's *κ* = 0.98). Results of this pilot phase are available in the Supporting Information. In the second phase, each author was randomly assigned approximately 50% of the remaining full‐text articles for independent screening and data extraction, with uncertainties resolved collaboratively.

### Conceptualization and operationalization of CBPR engagement

To capture variation in how CBPR was represented across studies, we developed a set of mutually exclusive, hierarchical study‐level codes reflecting increasing levels of engagement with CBPR: (a) mention, (b) discussion, and (c) use (see Table 2a). These categories distinguish between CBPR as an object of reference, a topic of substantive engagement, and a methodological approach guiding the research. Articles were classified into one of three categories based on the highest level of engagement observed:

1. **Mention**: Articles that referenced CBPR (e.g., in the Introduction or Discussion) without elaboration or application. These articles did not describe CBPR as informing the study design, methods, or analytic approach.
2. **Discussion**: Articles that engaged substantively with CBPR principles, frameworks, or implications but did not implement CBPR methods. This included theoretical discussion or calls for future use.
3. **Use**: Articles that explicitly implemented CBPR methods. To be classified as “use,” studies were required to describe CBPR as guiding the research design, procedures, or analytic approach, typically indicated by explicit reference in Section 2 and evidence of community involvement in one or more phases of the research process.

**TABLE 2a:** Categorization of community‐based participatory research (CBPR) engagement in manuscripts: mentioning, discussing, and using CBPR methods.

Category	Definition	
Mentioning CBPR	The manuscript refers to CBPR in a brief, passing manner without going into detail about its principles, processes, or implementation.	A single sentence or clause mentioning CBPR as part of a list of methodologies. A citation of CBPR in the introduction or background section without further elaboration.
Discussing CBPR	The manuscript provides a more detailed examination or discussion of CBPR, explaining its principles, benefits, challenges, or relevance to the topic at hand but does not report using it in the research.	A section of the manuscript is dedicated to explaining what CBPR is, its history, and theoretical background. A discussion on the potential application of CBPR in the context of the research, including its advantages and limitations.
Using CBPR methods or analysis	The manuscript reports on the actual application of CBPR principles and methods in the research process. This includes active participation of community partners in designing, conducting, and analyzing the research.	Descriptions of how community partners were involved in identifying the research questions, data collection, and data analysis. Reports on collaborative decision‐making processes and how community feedback influenced the research design and outcomes. Use of participatory data collection methods, such as focus groups or community surveys, where community members played a significant role.

This hierarchical coding scheme ensured that categories were mutually exclusive and reflected a graded continuum of engagement, from symbolic reference to full methodological implementation. Importantly, classification as a CBPR study (i.e., “use”) required explicit evidence of participatory methodology. Articles that only mentioned or discussed CBPR were not treated as CBPR studies in analyses focused on implementation. This approach allowed us to distinguish between the visibility of CBPR as an idea and its uptake as a methodological approach.

### CBPR elements of community engagement

For studies classified as using CBPR methods, we extracted nine elements of community engagement, including the nature of the researcher–community relationship, community input into research questions and design, collaboration on funding, oversight roles (e.g., ethics approval), participation in data collection and analysis, involvement in dissemination, integration of community priorities into the research aims, and indicators of sustainability (e.g., ongoing partnerships, data availability). These elements were selected and adapted from Collins and colleagues (2018, Table 1), whose framework contrasts traditional and participatory approaches across the research life cycle. Full coding criteria are presented in Table 2b.

**TABLE 2b:** Coding key elements of community‐based participatory research (CBPR) methods reported in studies.

Element	Description
Researcher–participant relationship	Manuscript mentions prior, long‐term, or developing relationship with community group(s).
2 Research idea or question	Community members involved in establishment of research question(s).
3 Funding	Community and researchers work together to secure funding; AND/OR Funding in place to ensure long‐term sustainability of relationships/interventions.
4 Oversight	Manuscript mentions ethics approval/oversight by a community group (CAB, tribal IRB, etc.).
5 Research/intervention design	Community input on design/methods; AND/OR community helps to design locally specific measures; AND/OR iterative processes, reflexivity, inductive methods, honoring subjectivity.
6 Data collection	Manuscript mentions community involved in data collection.
7 Data analysis	Manuscript mentions community perspectives inform analysis and/or interpretation of data.
8 Publication/dissemination	Manuscript mentions/has community members as co‐authors/owners; AND/OR dissemination of results in multiple formats (publications, fact sheets, websites, toolkits, etc.) with multiple ends (community education, policy, etc.).
9 Sustainability	Manuscript mentions long‐term sustainability (through availability of data to community for future use, continued relationships with community, etc.)

These elements were treated as observable indicators of community engagement rather than as a strict or exhaustive checklist. Coding was based primarily on information reported within each article. However, when authors referenced external publications (e.g., prior methodological papers or companion articles) for additional detail on CBPR procedures, these sources were consulted when clearly indicated and accessible. When additional detail was unavailable or could not be verified, coding was based on the focal article. As such, the coding strategy prioritized documented evidence of engagement while acknowledging that some studies may have implemented additional CBPR elements not fully described in the published report. This approach balances systematic documentation of participatory practices with variability in reporting across studies.

### Sample and study design characteristics

For studies meeting inclusion criteria, we extracted study‐ and sample‐level characteristics. These included (a) methodological approach (e.g., qualitative, quantitative, or mixed‐methods), (b) study design (e.g., observational, experimental, or longitudinal), (c) focal population (e.g., Black, Indigenous, multiracial participants, people living with HIV, or pregnant women), (d) primary research topic or substantive focus (e.g., medication adherence), and (e) geographic region of the sample (e.g., United States, Canada, or Nigeria).

## RESULTS

Across the 14,084 articles published in 10 health psychology journals between 2004 and 2024, 36 empirical studies (0.3%) met inclusion criteria by substantively engaging with CBPR. This indicates that CBPR remains minimally represented within the institutional core of health psychology.

**FIGURE 2:**
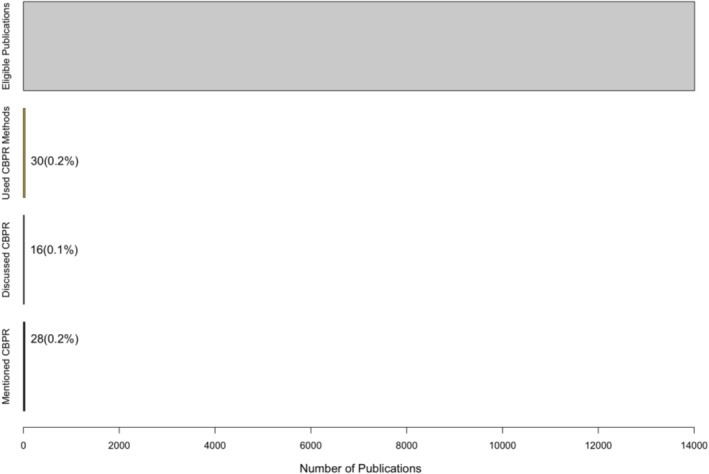
Community‐based participatory research engagement in 14,084 publications from 10 health psychology journals between 2004 and 2024.

Applying the graded classification scheme, studies varied in their level of engagement with CBPR. Of the 36 included studies, 6 (16.7%) were classified as mention, 0 (0.0%) as discussion, and 30 (83.3%) as use, reflecting explicit implementation of participatory methods. When contextualized relative to the full corpus of published articles, these correspond to approximately 0.04%, 0.00%, and 0.21%, respectively (see Figure 2). These findings indicate that CBPR appears infrequently in the selected journals and, when present, is more often implemented as a methodological approach than engaged as a conceptual or theoretical framework.

Table 3 summarizes sample, study, and methodological characteristics of the included studies, with detailed study‐level information provided in Table 4. Methodologically, the majority of studies employed qualitative approaches (20 studies, 55.6%), followed by mixed‐methods designs (nine studies, 25.0%) and quantitative approaches (seven studies, 19.4%). Study designs were predominantly observational (25 studies, 69.4%), with fewer intervention studies (seven studies, 19.4%), longitudinal designs (three studies, 8.3%), and a single case study (2.8%).

**TABLE 3:** Summary of sample, study, and methodological characteristics of studies meeting inclusion criteria (*N* = 36) and incorporating community‐based participatory methods (*N* = 30).

Characteristic	All studies	Studies using CBPR
Year		
2004–2009	3 (8.3)	2 (6.7)
2010–2014	10 (27.8)	8 (26.7)
2015–2019	7 (19.4)	6 (20.0)
2020–2024	16 (44.4)	14 (46.7)
Journal		
*British Journal of Health Psychology*	4 (11.1)	3 (10.0)
*European Journal of Health Psychology*	2 (5.6)	1 (3.3)
*Health Psychology*	5 (13.9)	4 (13.3)
*Journal of Health Psychology*	15 (41.7)	13 (43.3)
*Stigma and Health*	10 (27.8)	9 (30.0)
Method		
Mixed	9 (25.0)	9 (30.0)
Qualitative	20 (55.6)	16 (53.3)
Quantitative	7 (19.4)	5 (16.7)
Design		
Case study	1 (2.8)	1 (3.3)
Intervention	7 (19.4)	7 (23.3)
Longitudinal	3 (8.3)	3 (10.0)
Observational	25 (69.4)	19 (63.3)
Country^a^		
Australia	2 (5.6)	2 (6.7)
Cambodia	1 (2.8)	1 (3.3)
Canada	4 (11.1)	4 (13.3)
China	1 (2.8)	1 (3.3)
Croatia	1 (2.8)	0 (0.0)
Germany	1 (2.8)	0 (0.0)
Ireland	1 (2.8)	1 (3.3)
Nigeria	1 (2.8)	1 (3.3)
Papua New Guinea	1 (2.8)	1 (3.3)
Philippines	1 (2.8)	1 (3.3)
South Africa	2 (5.6)	2 (6.7)
Uganda	1 (2.8)	1 (3.3)
United Kingdom	5 (13.9)	4 (13.3)
United States	12 (33.3)	9 (30.0)
Zambia	2 (5.6)	2 (6.7)
Not specified	1 (2.8)	1 (3.3)
Population of focus^b^		
Chronic health conditions (e.g., people living with HIV)	9 (25.0)	9 (30.0)
High health risk populations (e.g., at‐risk youth)	2 (5.6)	2 (6.7)
Minoritized populations (e.g., sexual, ethnoracial)	13 (36.1)	10 (33.3)
Non‐vulnerable populations, including adults and adolescents	9 (25.0)	7 (23.3)
Vulnerable populations (e.g., underprivileged children)	4 (11.1)	3 (10.0)
Women	4 (11.1)	4 (13.3)
Health‐care stakeholders	2 (5.6)	1 (3.3)

^a^ One study included participants from Zambia and South Africa.

^b^ Some studies focused on multiple populations. See Supporting Information for coding criteria.

**TABLE 4:** Detailed summary of reports mentioning, discussing, or using community‐based participatory research (CBPR) methods and associated elements.

Year	Journal	Approach	Design	Population of interest	Primary research topic or substantive focus	Region of sample	Mentioned CBPR	Discussed CBPR	Used CBPR	Researcher–participant relationship	Research Question	Funding	Oversight	Research design	Data collection	Data analysis	Dissemination	Sustainability
2020	*European Journal of Health Psychology*	Quantitative	Observational	Law enforcement officers	Examines how health behaviors (e.g., sleep, exercise, pain) relate to subjective well‐being among police officers.	United States	X		X						X			
2024	*Stigma and Health*	Mixed	Longitudinal	Adults living and not living with HIV	Focuses on community‐level socio–structural dynamics shaping HIV stigma.	Zambia and South Africa	X	X	X	X			X		X	X		
2024	*Journal of Health Psychology*	Qualitative	Observational	At‐risk youth	Uses action research to engage marginalized youth in expressing their experiences and concerns.	Australia	X	X	X	X	X		X	X	X			
2019	*Journal of Health Psychology*	Mixed	Intervention	African American adults	Centers on a community‐based participatory intervention for cardiovascular health in African American communities.	United States	X	X	X	X	X		X	X	X		X	
2016	*Stigma and Health*	Qualitative	Observational	People living with HIV	Explores how people living with HIV/AIDS experience stigma, including self‐stigma vs. external stigma.	Canada	X	X	X					X	X		X	
2021	*Stigma and Health*	Mixed	Observational	Men who have sex with men	Explores the measurement and conceptualization of multidimensional sexual identity stigma.	South Africa			X	X				X				
2023	*Stigma and Health*	Quantitative	Observational	Adults with and without disabilities residing Intermountain West (Colorado, Utah, Idaho, or Wyoming)	Examines how discrimination amplifies the psychological impact of pandemic‐related stressors among people with disabilities.	United States	X											
2019	*Journal of Health Psychology*	Qualitative	Observational	Black lesbian adults	Investigates how intersectional identities shape health‐care experiences and self‐disclosure under minority stress.	United States	X											
2013	*Journal of Health Psychology*	Qualitative	Case study	Indigenous people of the Philippines	Investigates how members of an indigenous community conceptualize health.	Philippines	X	X	X									
2020	*Journal of Health Psychology*	Qualitative	Observational	UK parents	Examines what skills and strategies do parents use to communicate health information and manage their children's health.	United Kingdom	X	X	X				X		X	X		
2022	*Stigma and Health*	Mixed	Observational	Young adults	Uses concept mapping to identify how young adults conceptualize weight bias.	Canada			X							X		
2023	*British Journal of Health Psychology*	Mixed	Intervention	Adults with obesity (BMI ≥ 30 kg/m^2^; ≥ 27.5 kg/m^2^ for South Asian or African‐Caribbean individuals)	Develops and evaluates a behavioral intervention targeting weight loss maintenance, not just initial loss.	United States	X		X					X	X			
2015	*Journal of Health Psychology*	Qualitative	Intervention	16–25‐year‐olds living in South Wales, England	Uses participatory action research to co‐design an online support community.	England	X	X	X		X							
2024	*European Journal of Health Psychology*	Qualitative	Observational	Young (20–30‐years old), young– old (50–69 years old), and old–old (70 plus years old) adults	Examines age‐related variation in multidimensional views on aging in everyday life.	Germany	X											
2021	*British Journal of Health Psychology*	Qualitative	Observational	People with cancer diagnoses	Uses grounded theory to examine the cancer coping process.	Australia	X		X							X		
2010	*Journal of Health Psychology*	Quantitative	Intervention	Overweight Catholic Americans	Faith‐tailored obesity intervention within a Catholic community.	United States			X					X				
2010	*Journal of Health Psychology*	Mixed	Intervention	Women formerly selling beer in restaurants	Evaluates a participatory action research intervention (Hotel Apprenticeship Program).	Cambodia	X	X	X	X	X			X				
2012	*Health Psychology*	Mixed	Longitudinal	Asian American breast cancer survivors	Evaluates feasibility and preliminary efficacy of an expressive writing psychosocial intervention delivered via a CBPR framework	United States	X	X	X	X				X	X	X	X	
2008	*Health Psychology*	Quantitative	Observational	African American adults	Measurement and conceptualization of patient‐perceived health‐care provider cultural competency	United States	X	X										
2024	*Stigma and Health*	Qualitative	Observational	Men who have sex with men	Examines community member (MSM) involvement in an HIV stigma study using standardized patient methods.	China	X	X	X	X	X			X				
2011	*Journal of Health Psychology*	Qualitative	Observational	Irish women	Uses qualitative methods (interviews and focus groups) to examine beliefs, norms, and experiences surrounding alcohol use.	Ireland			X	X								
2023	*Stigma and Health*	Quantitative	Observational	Transgender and nonbinary adults	Investigates how rural upbringing shapes mental and physical health among transgender adults.	United States			X		X							
2021	*Journal of Health Psychology*	Quantitative	Longitudinal	Native Americans living with type 2 diabetes	Examines the bidirectional relationship between perceived glucose control and diabetes distress.	United States	X		X	X	X		X					
2022	*Stigma and Health*	Quantitative	Observational	Young adults living with HIV	Examines the differential effects of HIV stigma dimensions on health outcomes among youth	Zambia			X				X		X		X	
2010	*Journal of Health Psychology*	Qualitative	Observational	Young refugees	Focuses on contextual and participatory understanding of well‐being among refugee youth	England	X											
2014	*British Journal of Health Psychology*	Qualitative	Observational	Health‐care professionals and patients	Investigates organizational and relational determinants of health‐care quality across patient and provider perspectives.	Croatia	X											
2004	*Journal of Health Psycholog*y	Mixed	Observational	Adults impacted by plane crash disaster	Focuses on psychological and community impact of a large‐scale disaster on volunteers and communities	Canada	X	X	X	X			X	X		X	X	X
2010	*Health Psychology*	Qualitative	Observational	Older adults	Social representations of community and the role of community arts in social participation among older adults.	Britain	X		X	X								
2015	*Health Psychology*	Qualitative	Observational	Community workers	Narrative experiences of community health workers in community health psychology.	Britain	X		X									
2004	*Journal of Health Psychology*	Qualitative	Observational	Low‐income racial minorities	Examines how community residents (especially low‐income populations) participate in the planning and implementation of prevention programs	Canada	X	X	X	X	X		X		X			
2023	*Stigma and Health*	Qualitative	Observational	Sexual and gender minorities	Identifies and analyzes coping strategies used in response to stigma (e.g., avoidance, self‐monitoring, seeking support, cognitive reframing)	Nigeria			X							X		
2018	*Stigma and Health*	Qualitative	Intervention	People living with serious mental illness	Uses photovoice methodology to capture subjective experiences of stigma	United States	X	X	X						X	X	X	
2013	*Journal of Health Psychology*	Qualitative	Observational	African American and Latina women community health workers	Examines a PhotoPAR (participatory photography + action research) project with community health workers after Hurricane Katrina.	United States	X	X	X	X	X	X	X	X	X	X	X	
2024	*British Journal of Health Psychology*	Mixed	Intervention	Couples	Introduces a multi‐level, theory‐driven intervention to influence family planning behaviors	Uganda	X		X	X				X				
2015	*Health Psychology*	Qualitative	Observational	Adults with spinal cord injury and health‐care professionals working with this population	Use of narrative communication to translate and mobilize health knowledge into practice	Not specified			X					X				
2014	*Journal of Health Psychology*	Qualitative	Observational	Young adults	Examines youth participatory research (Photovoice) grounded in Freirean theory of critical consciousness.	Papua New Guinea	X	X	X	X			X		X	X	X	

**FIGURE 3:**
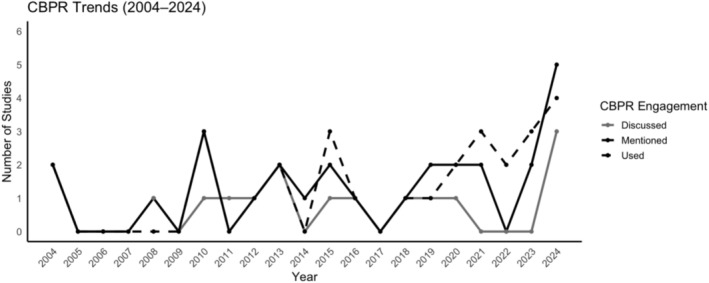
Community‐based participatory research trends (mentioned, discussed, implemented) from 2004 to 2024.

Temporally, CBPR‐related studies were concentrated in more recent years, with 16 studies (44.4%) published between 2020 and 2024. This pattern suggests a modest increase in the uptake of participatory approaches within the selected journals over time (see Figure 3).

In terms of journal distribution, CBPR‐related studies appeared most frequently in the *Journal of Health Psychology* (15 studies, 41.7%), followed by *Stigma and Health* (10 studies, 27.8%), *Health Psychology* (five studies, 13.9%), *British Journal of Health Psychology* (four studies, 11.1%), and *European Journal of Health Psychology* (two studies, 5.6%). Several journals in the sampling frame contained few or no CBPR studies, indicating variability in the uptake of participatory approaches across outlets.

Geographically, most studies were conducted in the United States (12 studies, 33.3%), followed by the United Kingdom (five studies, 13.9%) and Canada (4 studies, 11.1%). Ten studies (27.8%) were conducted in or focused on countries commonly classified as part of the Global South, including South Africa, Zambia, Uganda, Nigeria, Cambodia, the Philippines, and Papua New Guinea. Regarding populations of focus, studies most frequently examined minoritized groups, including sexual and ethnoracial minorities (13 studies, 36.1%), followed by individuals living with chronic health conditions such as HIV (nine studies, 25.0%), and general adult or adolescent populations (nine studies, 25.0%). Additional studies focused on populations experiencing structural vulnerability, including underprivileged children (four studies, 11.1%) and women (four studies, 11.1%).

Across the included studies, research topics clustered into several recurring domains. A substantial subset focused on stigma and related psychosocial processes, including its experience, measurement, and consequences, as well as coping responses and downstream health outcomes. A second domain centered on community‐engaged health interventions, including culturally tailored, faith‐based, and multi‐level approaches targeting outcomes such as cardiovascular health, obesity, and health behavior change. A third domain emphasized health literacy and communication, examining how individuals and communities access, interpret, and use health information within broader social contexts. Relatedly, several studies examined community participation and empowerment, focusing on how participatory processes (e.g., photovoice, action research) facilitate engagement and social change. Finally, a subset of studies addressed social and structural determinants of health, as well as psychological and relational processes shaping health, including coping, social norms, and identity‐related dynamics.

### Studies using CBPR methods

**FIGURE 4:**
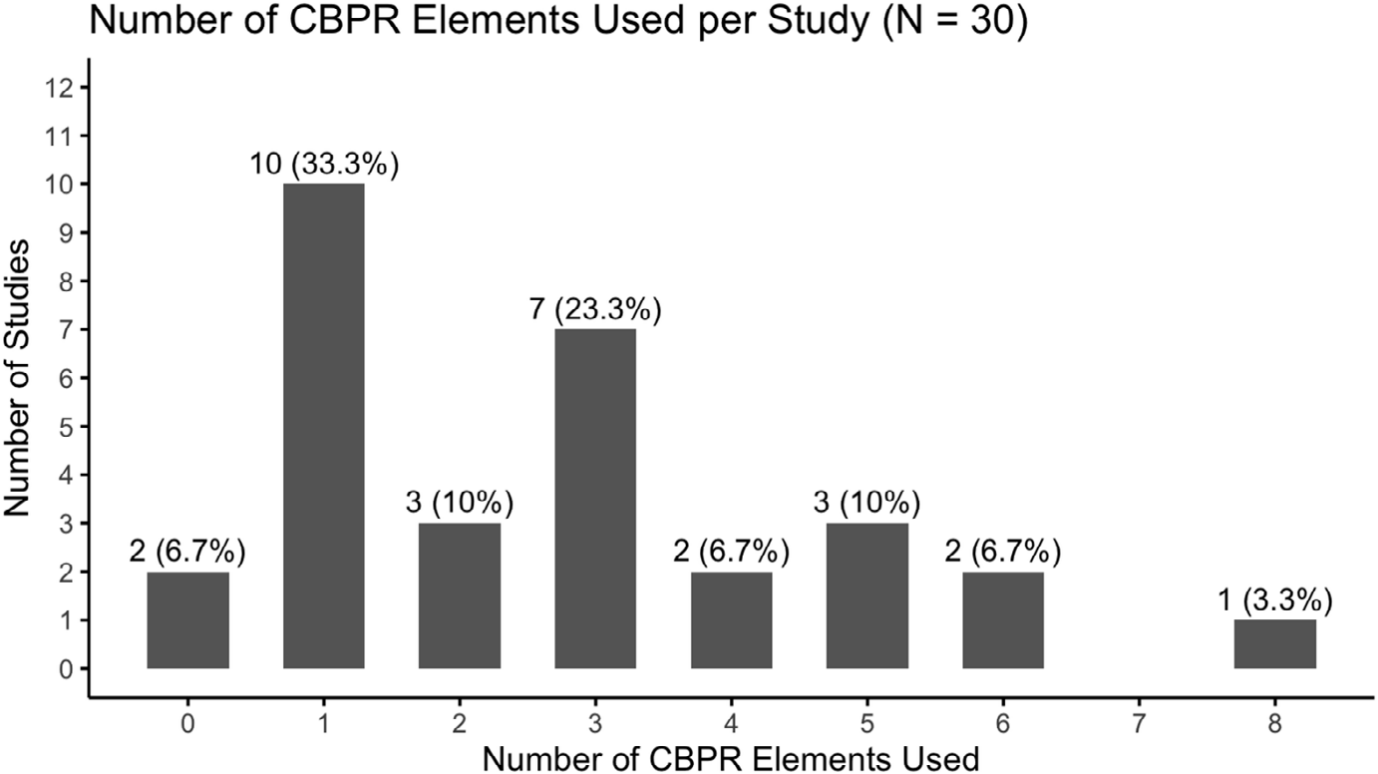
(a) Frequency and proportion of nine core community‐based participatory research (CBPR) elements across studies employing CBPR methods (*n* = 30). (b) Frequency and proportion of studies by the total number of community‐based participatory research elements addressed (*n* = 30).

As noted previously, 30 studies were classified as using CBPR methods (see Table 3 for a summary). In two cases, studies referenced companion methodological papers that provided additional detail on the participatory approach; consistent with our coding protocol, these sources were consulted to inform classification of CBPR engagement. Figure 4a displays the frequency and proportion of these studies that addressed each of the nine core CBPR elements (as defined in Section 2; see Table 2a). Engagement varied considerably: on average, studies incorporated 2.67 elements (*SD* = 2.99), with scores ranging from 0 to 8. The largest subset of studies (*n* = 10; 33.3%) addressed only one element. In total, 24 studies (80.0%) engaged with fewer than five elements, suggesting limited depth of engagement across much of the sample. At the high end, only one study (3.3%) reported addressing eight of the nine elements. Notably, two studies (6.7%) claimed to use CBPR methods but did not provide any details indicating engagement with the core elements. Figure 4b presents the distribution of studies by the total number of CBPR elements addressed, complementing the element‐specific data shown in Figure 4a.

Half of the studies (*n* = 15; 50.0%) described partnerships between researchers and community members, indicating some degree of sustained engagement. A smaller subset (*n* = 9; 30.0%) involved community stakeholders in the development or refinement of research questions. Community oversight, such as through advisory boards or tribal Institutional Review Boards (IRBs), was reported in 10 studies (33.3%). Thirteen studies (43.3%) described community involvement in the design of the study or intervention, as well as in data collection efforts. Slightly fewer studies (*n* = 10; 33.3%) included community members in the analysis phase, such as through collaborative interpretation of findings. Community participation in dissemination activities—such as co‐authorship or presenting findings in public forums—was reported in eight studies (26.7%). In contrast, very few studies included community members in funding‐related decisions, with only one study (3.3%) reporting shared budget control, collaborative grant writing, or financial planning. Likewise, only one study (3.3%) referenced concrete efforts to ensure sustainability beyond the life of the project, such as maintaining long‐term partnerships or providing continued data access.

## DISCUSSION

Considering the 2004 special issue that formally introduced community health psychology to the field (Campbell & Murray, 2004), we sought to examine whether CBPR is represented within the core publication outlets of health psychology. To address this, we systematically reviewed publications in 10 major health psychology journals over the past two decades. Our review identifies a central empirical pattern within these outlets: despite strong conceptual alignment with health psychology's equity‐oriented aims, CBPR remains minimally visible as a participatory research framework. Among ~14,000 articles published between 2004 and 2024, only 36 empirical studies (~0.3%) substantively engaged with CBPR. Of these, fewer than one in five clearly integrated CBPR principles across the full research life cycle. Overall, there has been a modest increase in CBPR‐related publications—particularly after 2015—although the overall uptake within these journals remains strikingly low. Given this pattern, our analysis focuses on studies that implemented CBPR methods, as only a small number of articles referenced CBPR without substantively incorporating its principles or practices.

Two journals accounted for about three quarters of these CBPR studies: *Journal of Health Psychology* and *Stigma and Health*. The prominence of *Journal of Health Psychology* may reflect the legacy of its 2004 special issue, which might have signaled openness to participatory approaches (Campbell & Murray, 2004). *Stigma and Health*, founded in 2016, has published more CBPR‐related papers than many established journals, likely because of its thematic focus and the influence of its inaugural editor (APA, 2022; Corrigan & Oppenheim, 2024). This pattern suggests that editorial positioning, thematic focus, and leadership may play a meaningful role in shaping the visibility of CBPR within core health psychology journals. Yet, overall, CBPR's presence within these outlets remains limited, reflecting broader disciplinary priorities and epistemic commitments (Rodriguez Espinosa & Verney, 2021).

A second pattern concerns the methodological profile of CBPR studies. About half of the CBPR‐engaged studies (53.3%) employed qualitative methods, consistent with CBPR's emphasis on co‐constructed knowledge (Grieb et al., 2015). However, the relatively infrequent use of mixed‐methods (30%) and limited use of quantitative designs (16.7%) suggests a constrained methodological range within these journals. Observational designs dominated across studies (63.3%), aligning with the exploratory aims of participatory research and also pointing to structural barriers that may constrain the implementation of more sustained or intervention‐focused designs (Goh et al., 2009; McFarlane et al., 2024). Longitudinal and experimental designs, which are critical for capturing change over time and evaluating impact, were less common. This relatively narrow methodological profile limits the scope and influence of CBPR within published health psychology research. Without greater pluralism in research design, the field risks missing opportunities to capture the full complexity of health inequities and to evaluate interventions in ways that reflect community priorities (Bishop, 2015; Cornish & Gillespie, 2009; McVittie, 2006). Greater methodological diversity would allow CBPR to contribute to multiple dimensions of rigor, including ecological validity, construct validity, and implementation relevance, particularly when paired with designs appropriate to the research question.

A third pattern is the uneven geographic distribution of CBPR research. One third of the studies were conducted in the United States, followed by the United Kingdom (13.3%) and Canada (13.3%). Countries in the Global South—including Nigeria, Uganda, Zambia, Cambodia, and the Philippines—were represented, but only minimally. This uneven distribution likely reflects disparities in research infrastructure, funding access, and institutional support for community‐engaged scholarship (Israel et al., 2017). At the same time, the limited presence of CBPR from the Global South within these publication outlets reinforces longstanding concerns about epistemic exclusion (Arnett, 2016; Openjuru et al., 2015). These disparities raise broader questions about whose knowledge is represented within the field's primary publication infrastructure and highlight the importance of expanding both the geographic scope and publication pathways for participatory research.

A fourth pattern concerns the populations with which CBPR is implemented. Slightly over a third of the studies focused on minoritized populations, whereas a substantial portion targeted broader populations not traditionally framed as vulnerable. Only a small number of studies focused explicitly on highly vulnerable groups, and just one engaged health‐care stakeholders. This distribution suggests a shift in how CBPR is being deployed within published health psychology research—from a historically justice‐oriented framework toward broader applications in population health (Rodriguez Espinosa & Verney, 2021). This evolution is not inherently problematic, but it raises important concerns. Without intentional integration of participatory principles into research design—not merely the selection of study populations—CBPR risks becoming disconnected from its justice‐oriented foundations (Collins et al., 2018; Suarez‐Balcazar et al., 2020; Wallerstein & Duran, 2010). As noted in Section 2, the elements coded in this review function as indicators of reported engagement rather than definitional criteria for CBPR, and variation in these elements reflects differences in implementation and reporting. When participatory principles are not deeply integrated across the research life cycle, CBPR may be interpreted more as a descriptive label than as a methodological approach grounded in shared authority and community partnership.

This issue is further reflected in the thematic concentration of CBPR across studies. Participatory approaches are clustered within a relatively narrow set of topics—most notably stigma, community‐based interventions, and health literacy—domains already defined by inequity, marginalization, and applied problem‐solving. Although this alignment is meaningful, it also indicates that CBPR remains underutilized in areas central to health psychology that focus on basic psychological processes, theory development, and mechanism testing (Giusto et al., 2024; Rodriguez Espinosa & Verney, 2021). These findings suggest that CBPR is often positioned as an intervention‐oriented or applied approach, rather than as a framework that can inform how constructs are defined, measured, and interpreted. As a result, opportunities may be missed to use participatory approaches to refine construct validity, identify context‐sensitive mechanisms, and generate ecologically grounded theory (Corrigan & Oppenheim, 2024; Giusto et al., 2024).

A final pattern concerns the depth of participatory engagement across studies. Despite modest growth in CBPR publications, many studies engaged communities primarily in early research phases, with more limited involvement in analysis, dissemination, or sustainability planning. Few studies incorporated core equity‐oriented practices such as shared decision‐making around funding or long‐term partnership development. This pattern suggests that CBPR within these journals is often implemented in partial or uneven ways (Collins et al., 2018; Israel et al., 2017). When participatory approaches are applied in a limited or instrumental manner, there is a risk that community engagement becomes procedural rather than transformative (Collins et al., 2018; Smith et al., 2010). Strengthening CBPR in health psychology therefore requires attention not only to whether participatory approaches are used but also to how fully they are integrated across the research process.

### Toward injecting CBPR values into health psychology research

Taken together, the limited visibility of CBPR within selected health psychology journals underscores the need for greater integration of participatory approaches within these outlets. Increasing the visibility of CBPR is critical for the field's intellectual and social progress, as participatory research aligns with justice‐ and health promotion‐oriented goals in the field. Despite these constraints, a complementary pattern is that CBPR‐informed interventions can yield measurable improvements in health outcomes. Systematic reviews have documented gains in participation, health behaviors, and health outcomes (Salimi et al., 2012; Yau et al., 2024). For example, Morisky et al. (2004) reported increased condom use and reduced STI incidence in a CBPR‐informed HIV prevention intervention among high‐risk heterosexual men in the Philippines. These findings demonstrate that participatory approaches can be implemented in ways that maintain methodological rigor while enhancing contextual relevance and intervention effectiveness.

The patterns identified in this review—limited uptake, uneven implementation, and structural constraints within core health psychology journals—point not only to gaps in the literature but also to opportunities for rethinking how CBPR can be more fully integrated into the field's primary publication outlets. Because this analysis is based on a bounded sample of journals, these interpretations speak specifically to patterns of representation within these outlets rather than the full distribution of CBPR research across health‐related disciplines, where participatory approaches are more widely established.

Although this review does not provide definitive explanations for CBPR's underrepresentation, the findings suggest several contributing factors. Some health psychologists may preferentially publish in community or public health journals perceived as more receptive. Others may lack training in CBPR or hold misconceptions about its rigor or feasibility (see Rodriguez Espinosa & Verney, 2021). In addition, prevailing peer review standards, funding priorities, and institutional reward systems tend to prioritize individual‐level, experimental, or quantitative approaches, potentially disincentivizing the collaborative, relational, iterative, and slow work that CBPR requires. Editorial preferences for methodological uniformity and generalizability may further constrain the acceptance of context‐specific, process‐oriented research.

Although a comprehensive guide to implementing CBPR is beyond the scope of this paper, existing resources provide detailed guidance (e.g., Fals‐Borda & Rahman, 1991; Israel et al., 2017; Marquez et al., 2022). To illustrate how CBPR principles can be enacted in practice, we highlight Scheib and Lykes (2013), which employed eight of the nine CBPR elements coded in this review. This study provides a concrete example of how participatory methods can be implemented across the research life cycle, from relationship‐building to dissemination. Rather than reiterating specific procedures, we draw attention to several general principles that characterize this approach.

First, Scheib and Lykes (2013) adopt an explicitly value‐oriented stance, articulating how their commitments shape the research process. Their project aims not only to document community experiences following Hurricane Katrina but also to enhance critical health literacy by foregrounding structural inequalities. This orientation requires treating community knowledge as epistemically coequal with researcher expertise, a principle that stands in contrast to dominant assumptions in mainstream health psychology.

Second, the study emphasizes process alongside outcomes. While the findings contribute to understanding health literacy in post‐disaster contexts, equal emphasis is placed on the relational and collaborative processes through which these findings emerge. Activities such as co‐training, shared dissemination, and sustained community engagement represent central outputs of the research itself. This contrasts with patterns observed in the present review, where community involvement was often concentrated in early phases rather than sustained across the research life cycle.

Third, the study demonstrates methodological flexibility. Although framed as a photovoice project, it incorporates multiple methods (e.g., photo elicitation, interviews, field notes, thematic analysis, collective drawing) and adapts these tools in response to the evolving research context. For example, the introduction of co‐researcher shadowing midway through the project reflects an iterative, in vivo development of method. This flexibility contributes not only to richer data but also to stronger community engagement. It also stands in contrast to the constrained methodological profile observed in this review, suggesting that greater methodological pluralism may be necessary for CBPR to be more fully represented within health psychology journals.

Although such flexibility may appear at odds with the standardization emphasized in post‐replication‐crisis psychological science, it need not be incompatible with open science practices. Rather, a productive integration may be possible, in which transparency and rigor are maintained alongside responsiveness to context and community. Developing this integration represents an important direction for advancing a form of community health psychology that is both methodologically rigorous and socially responsive.

### Constraints on generality and limitations

The conclusions of this review are bounded by the scope of the sampling frame and therefore should be interpreted as indexing the visibility and integration of CBPR within the institutional core of health psychology rather than its prevalence across health‐related research more broadly. The analysis was restricted to journals affiliated with major health psychology organizations, which represent a specific segment of the field's publication infrastructure. CBPR is more widely represented in adjacent disciplines, including public health, community psychology, and behavioral medicine, where participatory approaches are more firmly established. Accordingly, the patterns observed here reflect disciplinary uptake and publication practices within selected outlets, not the full distribution or impact of CBPR across domains of health research. In addition, variation in how CBPR is defined and enacted across contexts introduces heterogeneity that may not be fully captured by the present classification scheme, particularly in cases where participatory elements are embedded but not explicitly labeled as CBPR.

Several methodological limitations should also be considered. First, classification of CBPR engagement relied on information reported in published articles, which may incompletely reflect the extent of participatory processes, particularly given space constraints and variability in reporting practices. Although we consulted referenced methodological papers when available, some studies may have implemented additional CBPR elements that were not documented, leading to conservative estimates of engagement. Second, the hierarchical coding scheme, while designed to distinguish between levels of engagement, necessarily simplifies complex and iterative participatory processes into discrete categories. Third, the relatively small number of included studies limits the ability to draw strong conclusions about temporal trends, journal‐level differences, or population‐specific patterns. Finally, although inter‐rater reliability was high, coding decisions still involved interpretive judgment, particularly when assessing the presence and depth of participatory elements.

## CONCLUSION

This review provides a focused account of how CBPR is represented within the core publication outlets of health psychology, revealing that, over the past two decades, it remains limited in visibility, unevenly implemented, and structurally constrained despite its alignment with the field's commitments to equity, contextualized understanding, and the social determinants of health. As a framework grounded in community engagement, empowerment, and structural change, CBPR offers a means of advancing a form of health psychology that is both methodologically rigorous and socially responsive. Greater integration of participatory approaches has the potential to strengthen the cultural specificity, ecological validity, and practical relevance of research, while expanding the field's methodological and epistemological repertoire. Realizing this potential will require broader application of CBPR, as well as institutional and epistemic shifts within the field's publication infrastructure, including greater recognition of community expertise, support for equitable research partnerships, and evaluative standards that accommodate the relational and context‐sensitive nature of participatory work.

## CONFLICT OF INTEREST STATEMENT

The authors have no conflicts of interest to disclose.

## ETHICS STATEMENT

As the unit of analysis was papers, not individuals, and no personal data were collected, no informed consent or ethical approval was necessary.

## Supporting information


**Table S1:** PRISMA 2020 Checklist.

## Data Availability

The data that support the findings of this study are openly available in OSF at https://tinyurl.com/yc6v92ee.
